# Comprehensive analysis of the expression, prognosis, and immune infiltrates for CHDs in human lung cancer

**DOI:** 10.1007/s12672-022-00489-y

**Published:** 2022-04-25

**Authors:** Yang Lv, Wenchu Lin

**Affiliations:** 1 High Magnetic Field Laboratory, Hefei Institutes of Physical Science, Chinese Academy of Sciences, Hefei, 230031 Anhui People’s Republic of China; 2 University of Science and Technology of China, Hefei, 230026 Anhui People’s Republic of China; 3 Key Laboratory of High Magnetic Field and Ion Beam Physical Biology, Hefei Institutes of Physical Science, Chinese Academy of Sciences, Hefei, 230031 Anhui People’s Republic of China

**Keywords:** Lung cancer, CHD family, Prognosis, Bioinformatics analysis, Immune infiltrates

## Abstract

**Background:**

The chromodomain helicase DNA-binding (CHD) family, a group of genes that regulate nucleosome spacing and access to transcription factors, contributes to tumorigenesis in various cancers. However, the roles of CHD family members in lung cancer remain poorly understood.

**Methods:**

We investigated the transcriptional, survival, and immune data of CHDs in patients with lung cancer from the Oncomine, UALCAN, GEPIA, Kaplan–Meier Plotter, TCGA, TIMER, cBioPortal, and CR2Cancer databases. Then, perform functional enrichment analysis of CHDs was performed using the Metascape. Finally, the expression of CHD7, CHD8 and DNA damage response genes were evaluated by quantitative real-time PCR and western blot.The effects of CHD7 or CHD8 knockdown on A549 and PC9 cells were measured in vitro by flow cytometry, cell viability and colony formation assays.

**Results:**

We found that except for CHD5, nearly all members of CHDs in lung cancer showed altered expression compared with adjacent normal tissues. Moreover, the abnormal expression levels of CHDs were related to the clinical outcome of patients with lung adenocarcinoma and, to a lesser extent, patients with lung squamous cell carcinoma, which were significantly associated with the immune infiltrating levels of immune cells. Furthermore, the functions of CHDs and their neighboring genes are mainly related to DNA repair, the cell cycle, and organelle organization. Finally, cellular experiments conducted in vitro confirmed that CHD7/8 played indispensable roles in DNA damage signaling and cell cycle progression in lung adenocarcinoma cells.

**Conclusion:**

This study implied that CHD family members, especially in subclass III, are potential targets of precision therapy and new biomarkers for patients with lung cancer.

**Supplementary Information:**

The online version contains supplementary material available at 10.1007/s12672-022-00489-y.

## Introduction

Lung cancer remains the predominant cause of cancer-related deaths worldwide, making up almost 20% of cancer deaths [1]. Histologically, lung cancer is categorized into two main forms: small cell lung cancer (SCLC) and non-small-cell lung cancer (NSCLC). NSCLC accounts for approximately 85% of all lung cancers and can be further histologically divided into lung adenocarcinoma (LUAD), lung squamous cell carcinoma (LUSC), and large cell carcinoma. Lung cancer is often diagnosed at an advanced stage, as patients may experience no or minimal symptoms. Despite tremendous advances in lung cancer management in the past decade, the five-year relative percent survival rate among patients diagnosed with lung cancer remains approximately 19% [2]. Thus, the development of early-stage diagnosis and effective targeted therapy remains a significant clinical challenge in patients with lung cancer.

A plethora of mutations in epigenetic regulator genes identified recently by next-generation sequencing (NGS) highlights the importance of epigenetic dysregulation in cancer initiation, progression, and resistance to therapy [3]. In addition to abnormal histone or DNA covalent modifications that have been recognized as cancer drivers, recent studies indicate that chromatin remodeling is instrumental in tumorigenesis. The chromodomain helicase DNA-binding (CHD) family of enzymes is a subfamily of ATP-dependent chromatin remodeller complexes. CHD proteins can regulate nucleosome spacing and access to transcription factors using the energy derived from ATP hydrolysis. Moreover, inactivation of the CHD family has been implicated in various human cancers [4, 5]. The CHD family encompasses nine members (CHD1-9) subdivided into three subclasses according to their chromodomain types and the presence or absence of additional motif features [6]. Subclass I (CHD1 and CHD2) has a DNA-binding domain located in the C-terminal region [7]. Subclass II, which comprises CHD3, CHD4, and CHD5, contains dual plant homeodomains (PHDs) [8]. Subclass III (CHD6, CHD7, CHD8, and CHD9) has Brahma and Kismet domains with unknown functions [9].

Emerging evidence has revealed that subclass I and subclass II CHD proteins function as potential tumor suppressors, and their inactivation contributes to the development of a variety of cancers. Homozygous deletion of CHD1, the second most common genetic alteration in prostate cancer, could define a unique subtype of prostate cancer [10]. CHD2 has been proposed to prevent breast cancer initiation, and CHD2 mutations are associated with chronic lymphocytic leukemia. Among the subclass II members, CHD5 is the most well-studied protein and the first to be implicated in various cancers, including neural, epithelial, and hematopoietic malignancies [11]. At the same time, little evidence has shown alterations in CHD3 and CHD4 in human cancers. Furthermore, CHD family proteins have been implicated in drug resistance to therapy [12–14]. For example, CHD4 could regulate platinum sensitivity in the ovarian[12]. Unlike subclass I and II, subclass III CHD family genes have not been extensively investigated in cancer since they are more likely associated with developmental disorders and neurological syndromes in the clinic [13, 15–18]. Several recent reports suggest that CHD7, CHD8, and CHD9 are linked with cancers and show potential as biomarkers [19–21]. Although the dysregulation of CHD family genes has been examined and documented in a broad range of cancers, the roles of CHD-type chromatin remodellers in NSCLC, the predominant type of lung cancer, are poorly understood.

Therefore, the lack of association between CHD family genes and lung cancer prompts us to address the roles of CHD family genes to discover vulnerabilities and potential prognostic markers through integrative analysis of microarray, RNA-seq, and genomic profiling data using bioinformatics tools and molecular approaches. In this study, we performed a comprehensive analysis of the role of CHDs in lung adenocarcinoma and lung squamous cell carcinoma. In addition, we characterized the mutation landscape and expression patterns of CHDs and explored their potential prognostic values and biological functions. Finally, the potential biological roles of two CHD family members, CHD7 and CHD8, were illustrated in lung adenocarcinoma cells using experimental data.

## Material and method

### Data collection

#### TCGA-LUAD cohort and TCGA-LUSC cohort

The clinicopathological information and level 3 RNA-Seq data of CHD family genes of 1013 patients with LUAD and LUSC were available through the Cancer Genome Atlas (TCGA) portal [22] (https://tcga-data.nci.nih.gov/tcga). In addition, the visualized DNA methylation, expression, and clinical data were downloaded from MEXPRESS (https://mexpress.be) [23, 24]. Statistical significance was inferred at a *P* value < 0.05.

#### Oncomine database

Analysis of the mRNA expression data of CHD family genes in various cancers was performed by using the Oncomine database (https://www.oncomine.org) [25, 26]. Comparisons between the expression of CHD genes in lung cancer samples and that in normal controls were performed using a two-tailed Student’s t test. The cut-off of fold change and P value were defined as 1.5 and 0.01, respectively.

#### UALCAN dataset analysis

Differential expression analysis, prognostic analysis, and cancer type staging in LUAD and LUSC were carried out using a standard processing pipeline from UALCAN (http://ualcan.path.uab.edu), which is an interactive web resource for analysing cancer OMICS data [27].

#### Human protein atlas

Differential protein expression analysis between LUAD or LUSC and normal tissues was performed using the Human Protein Atlas (https://www.proteinatlas.org) [28], a proteomic resource that contains immunohistochemistry on tissue microarrays with 44 different tissue types.

#### cBioPortal

The mutation profiles of CHD family genes were analysed by a standard processing pipeline in the cBioPortal (https://www.cbioportal.org), a web-based database analysing multidimensional cancer genomics data. The lung adenocarcinoma dataset (TCGA, PanCancer Atlas) and lung squamous cell carcinomas (TCGA, PanCancer Atlas), which contained 507 and 469 pathological reports, respectively, were chosen for comprehensive genomic analysis [29, 30].

#### CR2Cancer database

The proportion of patients with different DNA copy number statuses in CHDs genes was determined using CR2Cancer (http://cis.hku.hk/CR2Cancer) [31], which is a comprehensive annotation and visualization database for CRs in human cancer constructed by high-throughput data analysis (e.g., TCGA and CCLE) and literature mining (PubMed).

### Survival association analysis

#### KM plotter database

The prognostic values of the expression of CHD family genes in patients with lung cancer were assessed using an online database, Kaplan–Meier plotter (www.kmplot.com). To analyse the OS and PFS of patients with lung cancer, patient samples were split into two groups by median expression (high versus low expression) and assessed by a Kaplan–Meier survival plot, with the hazard ratio (HR) with 95% confidence intervals (CIs) and log-rank p value [32].

### Immune microenvironment association

#### TIMER database

The TIMER (https://cistrome.shinyapps.io/timer) online system is a web server for comprehensive analysis of tumor-infiltrating immune cells. This study used a database to explore the association between clinical outcome and the abundance of immune infiltrates or CHDs gene expression [33, 34].

#### TISIDB database

TISIDB (http://cis.hku.hk/TISIDB) is a web portal for tumor and immune system interaction, which integrates multiple heterogeneous data types. It was used to analyse the relationship between CHDs mRNA expression in CHDs and the preimmune stage[35].

### Functional enrichment analysis

#### GEPIA dataset analysis

The correlations between the gene expression levels of the CHD family were analysed by using a standard processing pipeline in GEPIA (http://gepia.cancer-pku.cn), a newly developed interactive web server[36].

#### Metascape database

Gene Ontology and pathway enrichment analysis of CHD family genes and neighboring genes associated with CHD genes were performed using Metascape (https://metascape.org) [37].

### RNA interference

CHD7 and CHD8 siRNAs were designed and synthesized by General Biosystems (Hefei, China). Cells in good condition were seeded into 6-well plates at 50% density and transfected with CHD siRNAs or NC-siRNA using Lipo8000 transfection agent according to the manufacturer's protocol. After 48 h of culture, cells were collected for further analysis. The siRNA sequences were listed as follow:

Control siRNA: 5’- UUCUCCGAACGUGUCACGUTT -3’;

siCHD8#1: 5’- GGCACGAUGUCAUCGAAUUTT -3’;

siCHD8#2: 5’- GCAAGAUUCGGGAAUUUAATT -3’;

siCHD7#1: 5’-GCUGAUGACUGGAAGAAAUCG -3’;

siCHD7#2: 5’- GGAACAAGCCGAAGGCAAATT -3’.

### RNA isolation and RT–qPCR

Total RNA was extracted from lung cancer cells using a TransZol Up Plus RNA Kit (TRAN, ER501-01, China). Then, the RNA was converted into cDNA using the Evo M-MLV RT Mix Kit (Accurate Biotechnology, AG11728, China) following the manufacturer's instructions. Real-time PCR was performed by a Roche LightCycler 96 Real-Time PCR System. Actin was used as an internal control. The primers were as follows:

CHD7-F: 5’-TGATGAGTCTTTTTGGCGAGG-3’;

CHD7-R: 5’-CTGGATTTTCCGGGTAACCAC-3’;

CHD8-F: 5’-AAGCAAATCGGATTGTAGCAGA-3’;

CHD8-R: 5’-GGCAACTCGTCCTCATTTAAGA-3’;

Actin-F: 5’-TGTATGCCTCTGGTCGTACC-3’;

Actin-R: 5’-CAGGTCCAGACGCAGGATG-3’;

RAD51-F: 5’-CCTCCTCTTTAACGCCTCCTG-3’

RAD51-R: 5’-GGGGACAACTCCCAGACTTTTT-3’

### Cell cycle analysis

After knockdown of CHD family genes, the cells were fixed with EtOH at − 20 °C for 24 h. Fixed cells were then stained with PI/RNase staining buffer (BD Pharmingen, Franklin, NJ, USA). The percentage of cells in each cell cycle phase was determined using ModFit software (Verity Software House, Topsham, ME, USA).

### Western blot analysis

Cell lysates were prepared in lysis buffer (150 mM NaCl, 50 mM Tris–HCl, 1% Triton-X-100, 1 mM EDTA, EDTA-free PhosStop, and complete protease inhibitor mixture (Roche Applied Science)) on ice for 30 min. Subsequently, the cell debris was removed by centrifugation at 13,000 rpm for 10 min at 4 °C. The total protein concentration in the supernatant fluid was determined using a bicinchoninic acid (*BCA*) protein assay kit (Sangon Biotech, China). Finally, the proteins were denatured and then separated on polyacrylamide gels and reacted with specific primary and secondary antibodies. Proteins were visualized using an ECL chemiluminescence detection system. The antibodies used were as follows: γH2AX (1:1000, CST 2577), RAD51 (1:1000, Abcam ab133534), p-CHK1 (1:1000, Ser317, CST 12302), β-actin (1:1000, Transgen HC201-02), rabbit IgG (1:3000, CST 7074), mouse IgG (1:3000, CST 7076), p-RPA2 (1:1000, Ser4, Ser8, NBP1-23017), and CHK1 (1:1000, CST 2G1D5).

### Cell culture

The non-small-cell lung cancer cell lines A549 and PC9 were cultured in RPMI-1640 complete medium containing 10% fetal bovine serum and 1% penicillin/streptomycin at 37 °C in a humidified atmosphere of 95% air and 5% CO_2_.

### Cell viability assays

At 48 h posttransfection, cells (3000 per well) were seeded into 96-well plates and cultured for 72 h. Cell viability was assessed by the CellTiter-Glo Luminescent assay (Promega, USA), and luminescence was recorded using an Envision PerkinElmer porous plate microplate reader.

### Statistical analysis

All in vitro analyses were repeated at least three times. P values < 0.05 were considered statistically significant. The quantitative results were analysed by two-tailed unpaired Student’s t tests in GraphPad Prism software.

### Colony formation assay

After knockdown treatment, the cells were cultured in a six-well plate until the clone size was appropriate (more than 10 days), and then the medium was removed and cleaned with PBS. Methanol was used to fix the cells for 1 min, sucked out and 1 ml 0.1% crystal violet solution was added to each well. Finally, the samples were dyed for 15 min, rinsed, dried and photographed.

### In vitro EdU incorporation assay

EDU staining was performed using the BeyoClick™ EdU Cell Proliferation Kit with Alexa Fluor 488 (Beyotime). After knockdown of CHD7/8, the cells were cultured overnight in a six-well plate. Then EDU dye was added to the six-well plate until the final concentration reached 10uM. Cells were collected after 2 h of culture, which were fixed with 4% paraformaldehyde. After 15 min of fixation, the cells were permeated with 0.3% Triton X-100. Finally, the cells were stained with dye solution, sealed,and photographed.

### Drug dose response curves

Cells transfected siControl, siCHD7#1, or siCHD8#1 were cultured in 96-well plates overnight and treated with different doses of cisplatin or paclitaxel for 24 h. Then assessed cell viability and used an IC50 model of GraphPad to determine IC50 values.

## Results

### Altered expression of CHDs in patients with lung cancer

Nine different CHD family members are expressed in mammalian cells. The transcriptional levels of nine CHDs in 20 different types of cancer diseases were determined using the Oncomine database. The results revealed that CHD4/6/7/8 was significantly upregulated in multiple datasets in lung cancer compared with normal tissues. Meanwhile, CHD1/2/3/9 were downregulated in lung cancer (Fig. S1A, p < 0.01, F > 1.5)**.** Specifically, the gene expression level of CHD4 was significantly upregulated in three data cohorts (Stearman Lung, Fold Change = 1.513; Hou Lung, Fold Change = 1.744; Selamat Lung, Fold Change = 1.75). Similarly, the expression level of CHD5 was upregulated in two subtypes of lung cancer, lung carcinoma (fold change = 8.841) and small cell lung carcinoma (fold change = 6.562). Compared to normal samples, CHD7 was overexpressed in large cell lung carcinoma with a fold change of 2.741, papillary lung adenocarcinoma with a fold change of 1.162, and lung adenocarcinoma with a fold change of 1.491. The expression level of CHD8 was upregulated in Selamat Lung (fold change = 1.847), whereas the expression level of CHD9 was uniformly downregulated in four data cohorts (Yamagata Lung, fold change = − 1.451; Yamagata Lung, fold change = − 1.43; Landi Lung, fold change = − 1.404, Okayama Lung, fold change = − 1.19) (Table. S1).

**Fig. 1:**
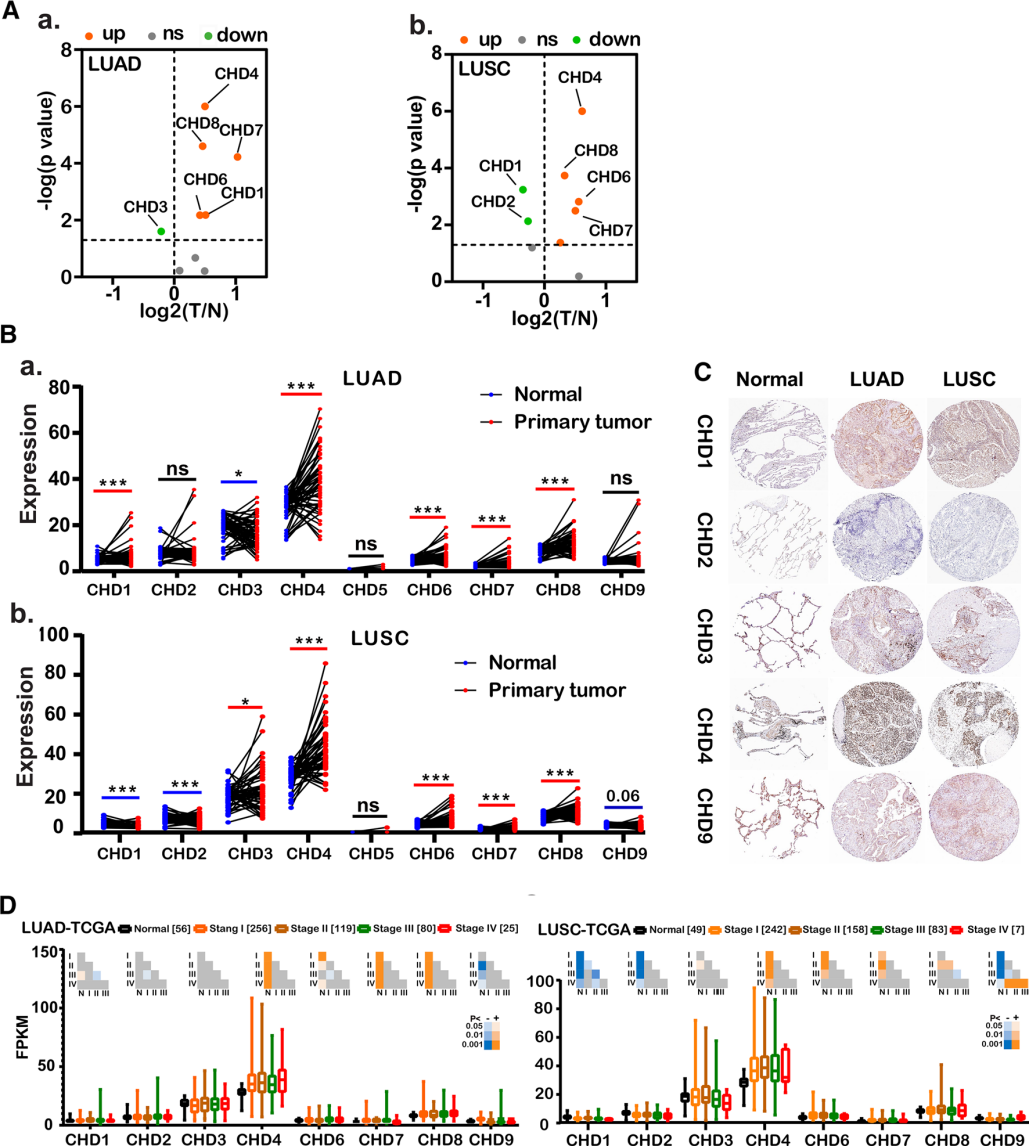
Aberrant expression of CHDs in lung cancer. **A** Vocano plots illustrating differential changes in CHDs in lung adenocarcinoma versus normal tissues from TCGA database; **b** Vocano plots illustrating differential change of CHDs in lung squamous cell carcinoma versus normal tissues from TCGA database. Values are presented as log2. Gray, p > 0.05; Green, p < 0.05; Orange, p < 0.05;. T, primary tumor. N, normal tissue. LUAD, lung adenocarcinoma, LUSC, lung squamous cell carcinoma. **B a** mRNA expression of CHDs in 56 paired lung adenocarcinoma and adjacent normal tissues from the TCGA database; **b** 49 paired lung squamous cell carcinoma and adjacent normal tissues from the TCGA database. *p < 0.05, **p < 0.01, ***p < 0.001. **C** Representative immunohistochemistry images of CHDs in lung cancer and normal lung tissues (Human Protein Atlas). Left panel, normal lung tissues. Middle panel, LUAD. Right panel, LUSC. **D** Boxplot showing the association between CHD expression and tumor stages

To investigate the deregulation of CHDs in lung adenocarcinoma (LUAD) and squamous cell carcinoma (LUSC), the two main subtypes of lung cancer, the mRNA expression of CHDs was further analyzed using the UALCAN portal, which is an interactive tool for analyzing TCGA RNA-sequencing data. The results showed that the expression levels of CHD4/6/7/8 in LUAD and CHD3/4/6/7/8 in LUSC were significantly elevated, whereas CHD9 in LUAD and CHD1/2/9 in LUSC were decreased compared with normal tissues (Fig. S2B, C). Furthermore, the RNA-sequencing data of 56 paired LUAD and matched adjacent non-tumor tissues and 49 paired LUSC and matched adjacent non-tumor tissues were further analyzed. As shown in Fig. 1A_a/b, B_a/b, consistent with the observations from Oncomine data and UALCAN analysis, the expression of CHD4/6/7/8 was significantly upregulated in both LUAD and LUSC. In contrast, the mRNA levels of CHD1 and CHD3 in LUAD presented the opposite tendency to that in LUSC.

We next evaluated the relationship between the mRNA expression levels of CHDs and the different clinicopathological subgroups of patients in the TCGA lung cancer cohort. As shown in Fig. 1D, the upregulated mRNA expression levels of CHD4/7 were significantly associated with high cancer stages in LUAD. These features were also observed in LUSC, although to a lesser extent. Interestingly, unlike LUAD, the decrease in CHD1/2 mRNA expression levels in LUSC was significantly associated with cancer stage. Then, protein is the primary carrier of gene function. Furthermore, a comparative investigation of the protein expression patterns of CHDs in lung cancer was performed using annotated images from the Human Protein Atlas. Similar to the transcriptional alterations, the protein levels of CHD1 and CHD4 were remarkably higher in LUAD, and the proteins of CHD2 and CHD9 were expressed at lower levels in LUAD than in normal tissues. However, the expression of CHD3 was low, contrary to the change in RNA levels. Similarly, CHD2, CHD3, CHD4, and CHD9, but not CHD1, were consistent with the RNA expression levels in LUSC (Fig. 1C). In short, these findings indicate that the expression of CHD family members is associated with patients with different histological types of lung cancer and correlated with the tumor stages of patients with lung cancer, suggesting that CHDs might play significant roles in the tumorigenesis and progression of lung cancer, particularly LUAD and LUSC.

### Genetic alterations of CHDs in lung cancer

**Fig. 2:**
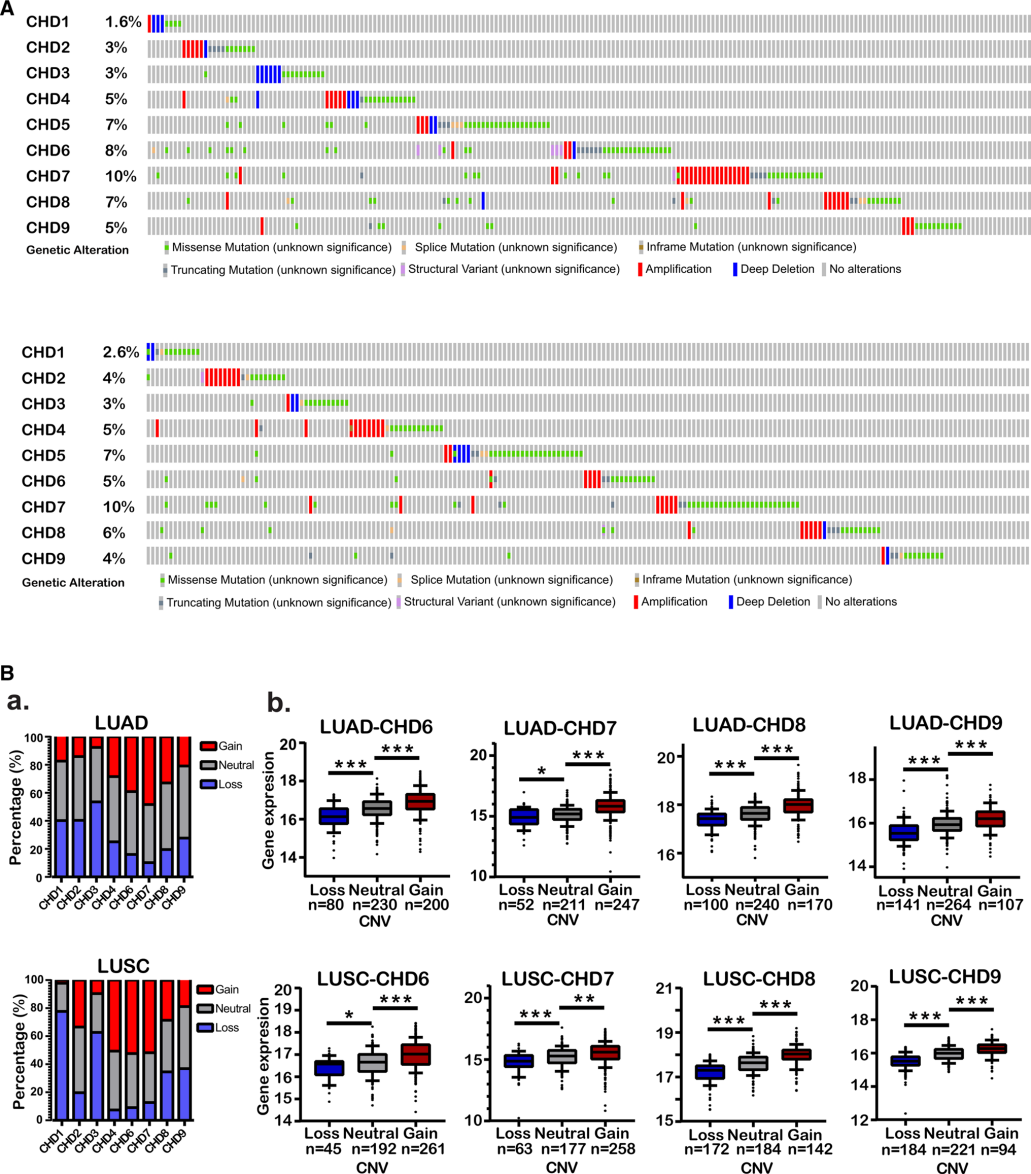
The association of CHD expression with genetic alterations in lung cancer. **A** OncoPrint showing the distribution and proportion of samples with genetic alterations in CHD genes in LUAD and LUSC; **B a** The contribution of copy number variation to gene expression variation in LUAD and LUSC; **b** Left panel, the proportion of samples with copy number variations for each CHD gene in LUAD or LUSC. Right panel, the correlation of copy number variation and CHD expression

Genetic alterations that occur in coding regions could influence gene expression and might contribute to tumorigenesis. Therefore, we analyzed the somatic mutation spectrum of CHDs by using the cBioPortal online tool for LUAD and LUSC. CHD genes were altered in 188 samples of 507 patients with lung adenocarcinoma (37%). Two or more alterations were detected in almost 1/4 of the samples (Fig. 2A). Notably, CHD5, CHD6, CHD7, and CHD8 were the four most frequently altered genes (7%, 8%, 10%, and 7%, respectively). Similarly, in the 469 sequenced LUSC patients, genetic alterations were found in 177 LUSC patients, and the total mutation rate was 37% (Fig. 2A). To address whether genomic structural variations, especially DNA copy number variation (CNV), play significant roles in modulating the expression of CHDs, the CR2Cancer and MEXPRESS online tools were applied to explore the transcriptional consequences of CHD CNV. First, the DNA copy number statuses of CHDs were characterized by the CR2Cancer database. The results showed a significantly greater percentage of copy number losses of CHD1/2/3 in LUAD and CHD1/3/8/9 in LUSC. In contrast, copy number gains of CHD 4/6/7 were commonly observed in both LUAD and LUSC (Fig. 2B_b). MEXPRESS online tool was then used to elucidate the association between CHD CNV and RNA expression in lung cancer. Analysis of over 1000 lung cancers of known copy number of CHD 6/7/8/9 showed a clear correlation between CNV state and transcript expression in LUAD and LUSC (Fig. 2B_b, Fig. S1F). CHD 1/2/3/4 also showed similar patterns but to a lesser extent (Fig. S1D, E). These results implied that CHD genes were mutated infrequently, and the deregulation of CHDs might be partly due to copy number variation in LUAD and LUSC.

### Prognostic values of CHD genes in patients with lung cancer

**Fig. 3:**
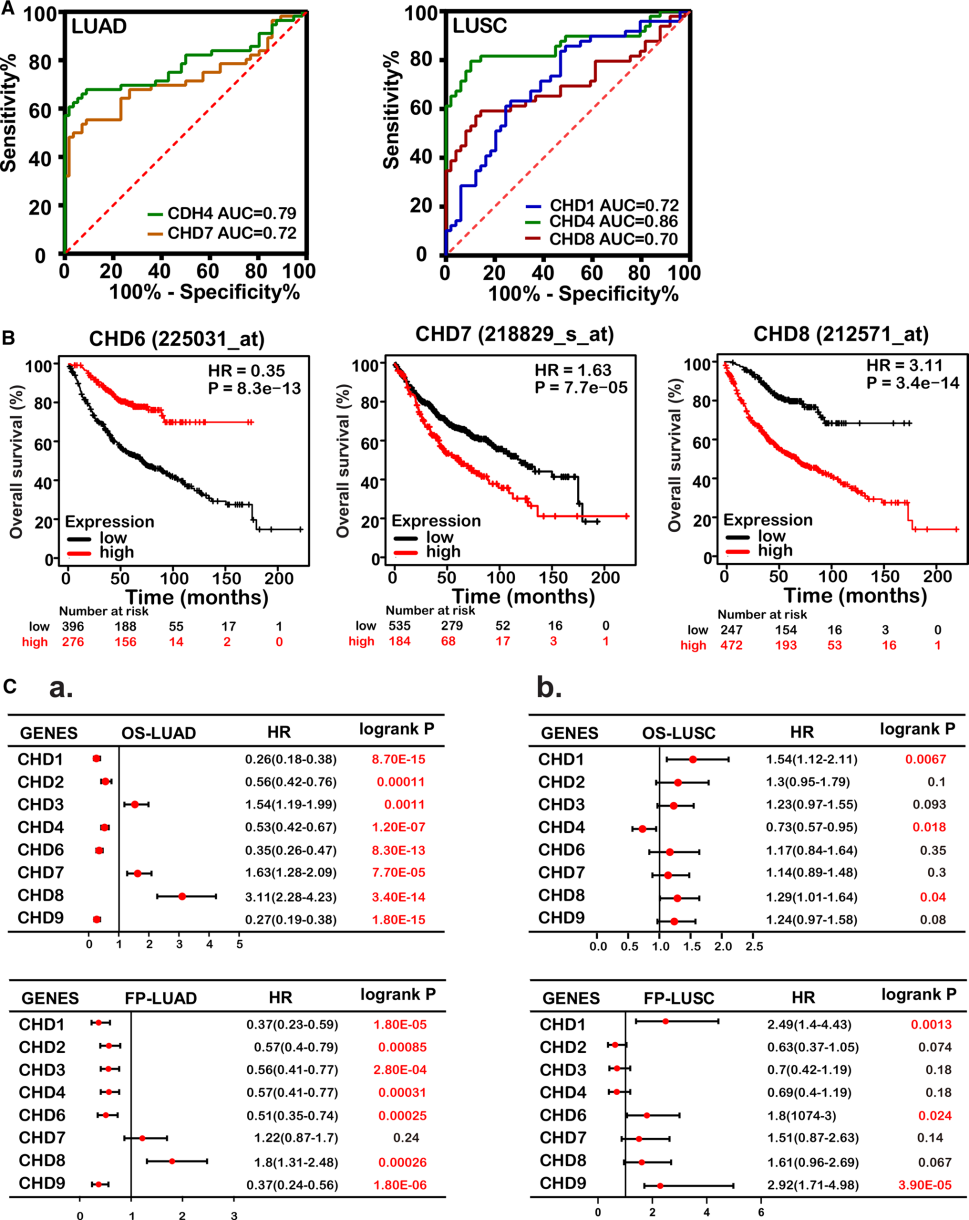
The correlation between CHD expression and clinical prognosis in patients with lung cancer. **A** ROC analysis of CHD expression for distinguishing lung cancer from normal lung tissues. left panel, LUAD; right panel, LUSC; **B** Kaplan–Meier analysis showing that expression of CHD6/7/8 was linked to overall survival in LUAD; **C a** Forest plots showing the association between the mRNA expression levels of CHDs and overall survival in LUAD and LUSC; **b** Forest plots showing the association between the mRNA expression levels of CHDs and first progression survival in LUAD and LUSC

Receiver operating characteristic (ROC) curves were plotted to determine the diagnostic efficiency of CHD expression in discriminating lung cancer patients from healthy individuals. The data showed that CHD4 had the highest area under the curve (AUC = 0.79). CHD7 had the second-highest AUC score (0.72). The AUCs of other CHDs, including CHD1, CHD2, CHD3, CHD6, CHD8, and CHD9, were lower than 0.70 in LUAD (Fig. 3A, Fig. S1G). The diagnostic sensitivity of CHDs was also assessed in LUSC. As shown in Fig. 3A and Supplementary Fig. 1H, transcriptional levels of CHD1/4/8 were associated with greater diagnostic accuracy. Among them, the AUC value of CHD4 had the highest AUC value, which was 0.86 with a sensitivity of 79.6% and specificity of 89.8%. To date, the potential prognostic value of CHD family members remains largely unknown. Therefore, we analyzed the correlation between the mRNA levels of CHDs and the survival of patients with lung cancer by using publicly available datasets. The patients were separated into high and low expression groups based on the cut-off value. Kaplan–Meier (KM) curve analyses showed that all CHD family members were significantly associated with the overall survival (OS) of LUAD patients. Among these genes, CHD3, CHD7, and CHD8 were risk genes, with a hazard ratio (HR) of > 1 and a log-rank P value of < 0.05. Conversely, CHD1, CHD2, CHD4, CHD6, and CHD9 were protective genes, with an HR of < 1 and a log-rank P value of < 0.05 (Fig. 3B, 3C_a, Fig. S2C). In contrast, only CHD1/4/8 was significantly correlated with the OS of LUSC patients (Fig. 3C_b, Fig. S2D). Moreover, almost all CHD genes correlated with the first progression survival (FP) of LUAD patients (Fig. 3C_a, Fig. S2C), while only CHD1/6/7/9 was significantly associated with the FP of LUSC patients (Fig. 3C_b, Fig. S2D). These results indicated that transcriptional expression of CHD family genes might be helpful in the prognosis of NSCLC patients, even though CHD2/3/6/7/9 were not independent predictors of OS and CHD2/3/4/8 were not independent predictors of FP in LUSC patients.

### Correlations between CHD family genes and immune infiltration levels

**Fig. 4:**
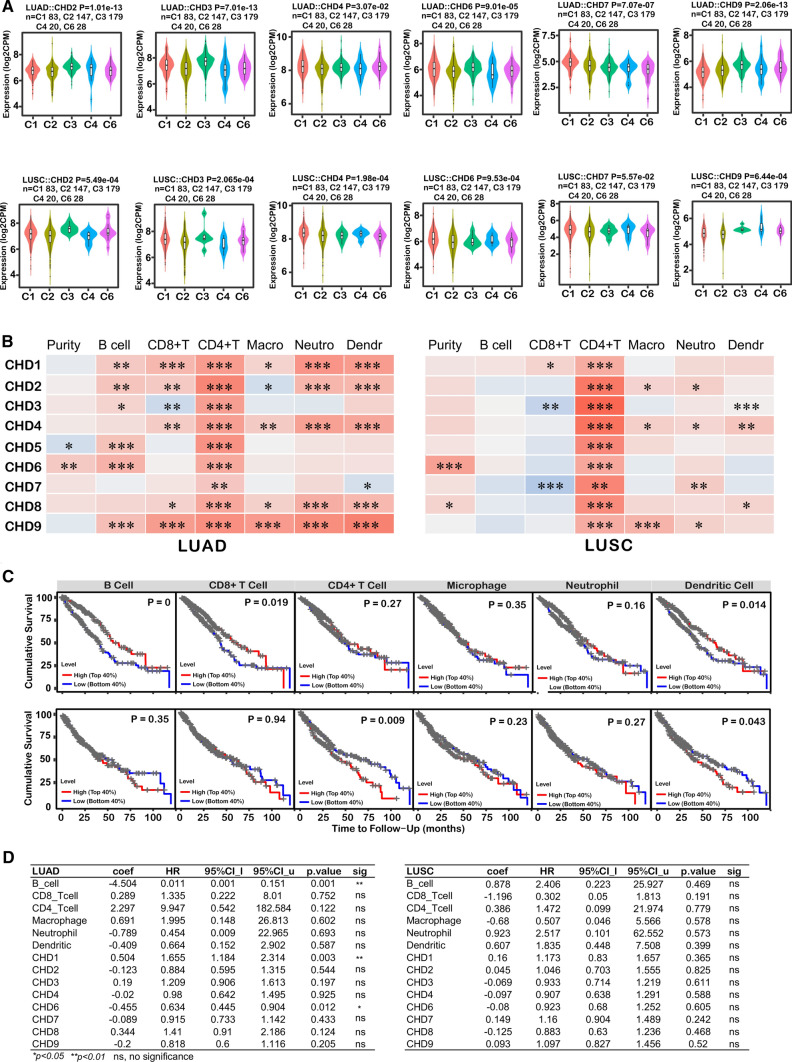
The association of CHDs with immune infiltrate levels in lung cancer. **A** The relationship between CHD expression and immune subtypes in LUAD (up) and LUSC (down); **B** The association between CHD expression and immune infiltration levels in LUAD (left panel) and LUSC (right panel); **C** Kaplan–Meier analysis of immune infiltrate levels in patients with lung cancer. Top panel, LUAD; bottom panel, LUSC; **D** The Cox proportional hazard model of eight CHD genes and six tumor-infiltrating immune cells in patients with lung cancer. Left panel, LUAD; right panel, LUSC

The TISIDB online tool was applied to explore the relationship between mRNA expression levels of CHDs and immune subtypes of NSCLC patients. It was evident from the illustration in Figure**.** As shown in Fig. 4A, CHD2, CHD3, CHD4, CHD6, and CHD9 were significantly associated with immune subtypes of both LUAD and LUSC. Meanwhile, CHD7 was only related to the immune staging of LUAD (Fig. S3A & Fig. S3B). Next, we wondered whether the expression of CHDs was involved in immune cell infiltration, which might affect the prognosis of lung cancer patients. Correlation analysis using the TIMER database showed that CHD1/2/4/8/9 expression was positively correlated with the infiltration of CD8 + T cells, CD4 + T cells, macrophages, neutrophils, and dendritic cells, and CHD1/2/3/5/6/9 was positively correlated with B cell infiltration in LUAD. Notably, CHD3 was positively associated with the infiltration of CD4 + T cells, macrophages, neutrophils, and dendritic cells and negatively correlated with CD8 + T cell infiltration in LUAD. Similarly, all CHD family members were strongly linked to CD8 + T cell infiltration in LUSC. However, CHD family members displayed weak or no correlation with the infiltration of other immune cells in LUSC (Fig. 4B, Fig. S3C & Fig. S3D). As shown in Fig. 4C, the abundance of infiltrating B cells, CD8 + T cells, and dendritic cells was significantly positively correlated with the clinical prognosis of LUAD patients. In contrast, the degree of infiltration of CD4 + T cells and dendritic cells was significantly negatively associated with overall survival in LUSC (Fig. 4C). In summary, it was apparent that CHD family genes played indispensable roles in the lung cancer immune microenvironment, which likely affects the survival of LUAD and LUSC by influencing the level of immune cell infiltration. To further explore the role of the CHD family in tumor immune infiltration, B cells, CD8 + T cells, CD4 + T cells, macrophages, neutrophils, and CHD1/2/3/4/6/7/8/9 cells were used to construct a Cox proportional hazards model using TIMER online tools. The results showed that the infiltration of B cells (P = 0.001) and the expression of CHD1 (P = 0.003) and CHD6 (P = 0.012) were significantly correlated with the clinical outcomes of patients with LUAD (Fig. 4D). Unfortunately, we did not find a significantly correlated variable factor in LUSC (Fig. 4D).

### Functional enrichment analysis of the CHDs family in patients with non-small-cell lung cancer

**Fig. 5:**
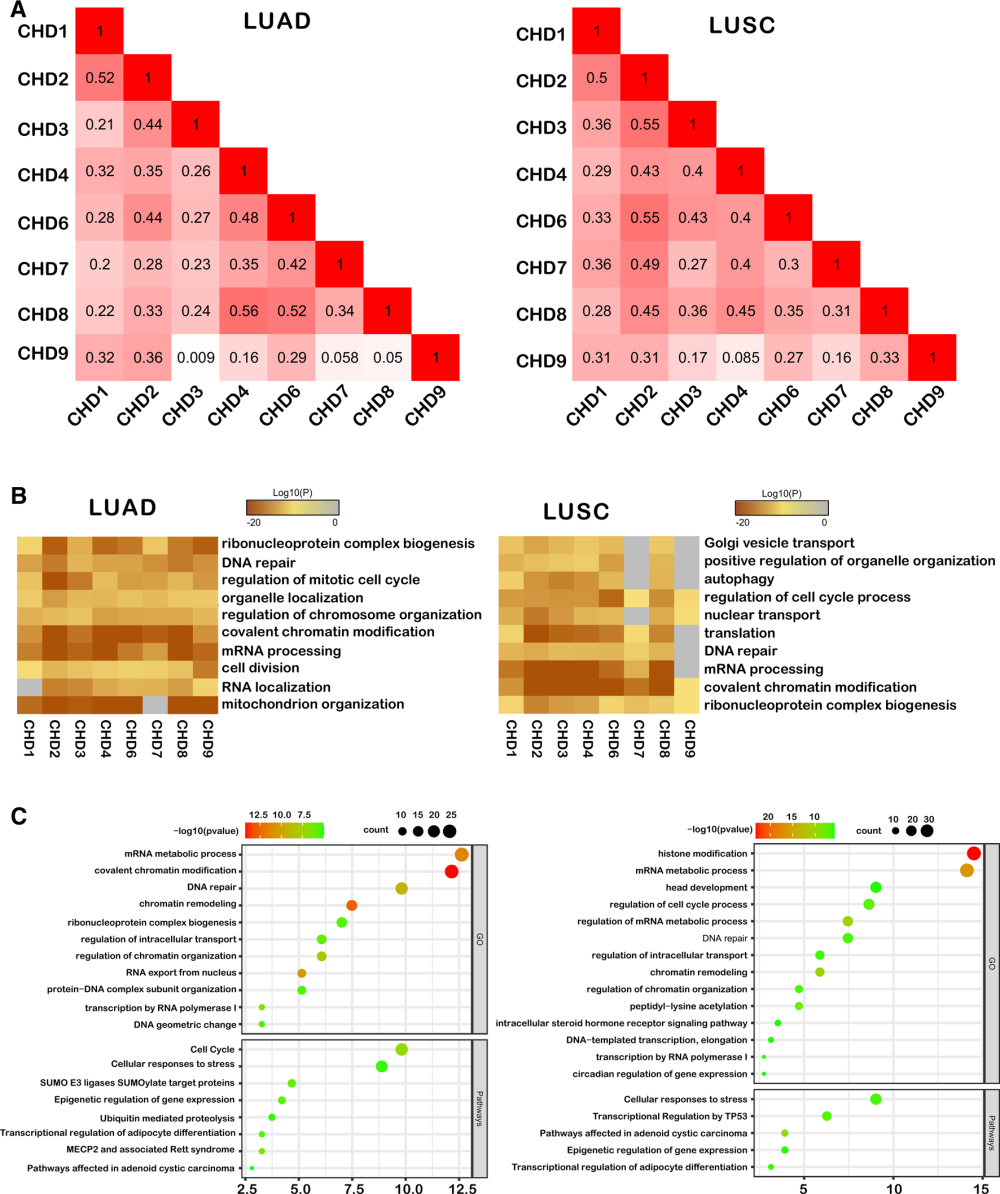
Functional enrichment analysis of CHDs in patients with lung cancer.** A** heatmap showing the correlation between different CHDs in LUAD (left panel) and LUSC (right panel); red cells indicate co-occurrence; The numbers in the color blocks represent the r-values; **B** Gene Ontology enrichment analysis of CHD co-expression genes. LUAD: |r|> 0.4, LUSC: |r|> 0.4; **C** Bubble diagram displaying the GO and KEGG functional enrichment results of CHD7 co-expression genes (left panel) and CHD8 co-expression genes (right panel). LUAD: |r|> 0.4, LUSC: |r|> 0.35

To dissect the function of CHDs in non-small-cell lung cancer, we first explored the potential co-expression genes of CHDs using the GEPIA database. Pearson’s correlation analysis revealed significant correlations in the following CHDs: CHD1 with CHD2/3/4/6/7/8/9; CHD2 with CHD3/4/6/7/8/9; and CHD3 with CHD4/6/7/8 and CHD4 with CHD6/7/8; and CHD6 with CHD7/8/9; and CHD7 with CHD8 in LUAD and LUSC. Differently, CHD9 and CHD7/8 showed a correlation in LUSC (Fig. 5A). These results indicated that CHDs interact with each other during the pathogenesis of lung cancer. Then, a GGI network for CHDs and the genes that strongly interact with the CHDs family in shared protein domains, physical interactions, co-expression, and pathways were constructed using GeneMANIA online tools. As seen from the Supplementary Fig. 4A, CBXs family were closely associated with CHDs. Notably, C17orf64 has the highest correlation score with CHD1 and CHD2. Next, we selected genes that were coexpressed with CHDs for functional enrichment analysis using Metascape online tools. The selected GO analysis results showed that ribonucleoprotein complex biogenesis, DNA repair, regulation of the cell cycle, organelle organization, covalent chromatin modification, and mRNA processing were highly related to the expression of CHDs in both LUAD and LUSC (Fig. 5B). Then, we selected CHD7 and CHD8, two members of subclass III whose biological function remains largely unknown in lung cancer, for subsequent pathway and function enrichment in LUAD and LUSC.

In LUAD, we selected genes common to 2 groups (|r|> 0.4) and found that CHD7 and CHD8 were related to DNA repair after GO analysis and to the cell cycle after Reactome Gene Sets analysis. In LUSC, we selected genes common in 2 groups (|r|> 0.35), which are related to the regulation of the cell cycle process, DNA repair, and other pathways (Fig. 5C). Subsequently, we performed GO analysis on related genes of the CHD family, which was similar to the enrichment analysis results by the Metascape. In particular, in LUAD, the CHD family was associated with DNA damage and DNA repair, which we focused on identification by cellular experiments.

### Chd7/8 loss affects cell proliferation and cycle progression

**Fig. 6:**
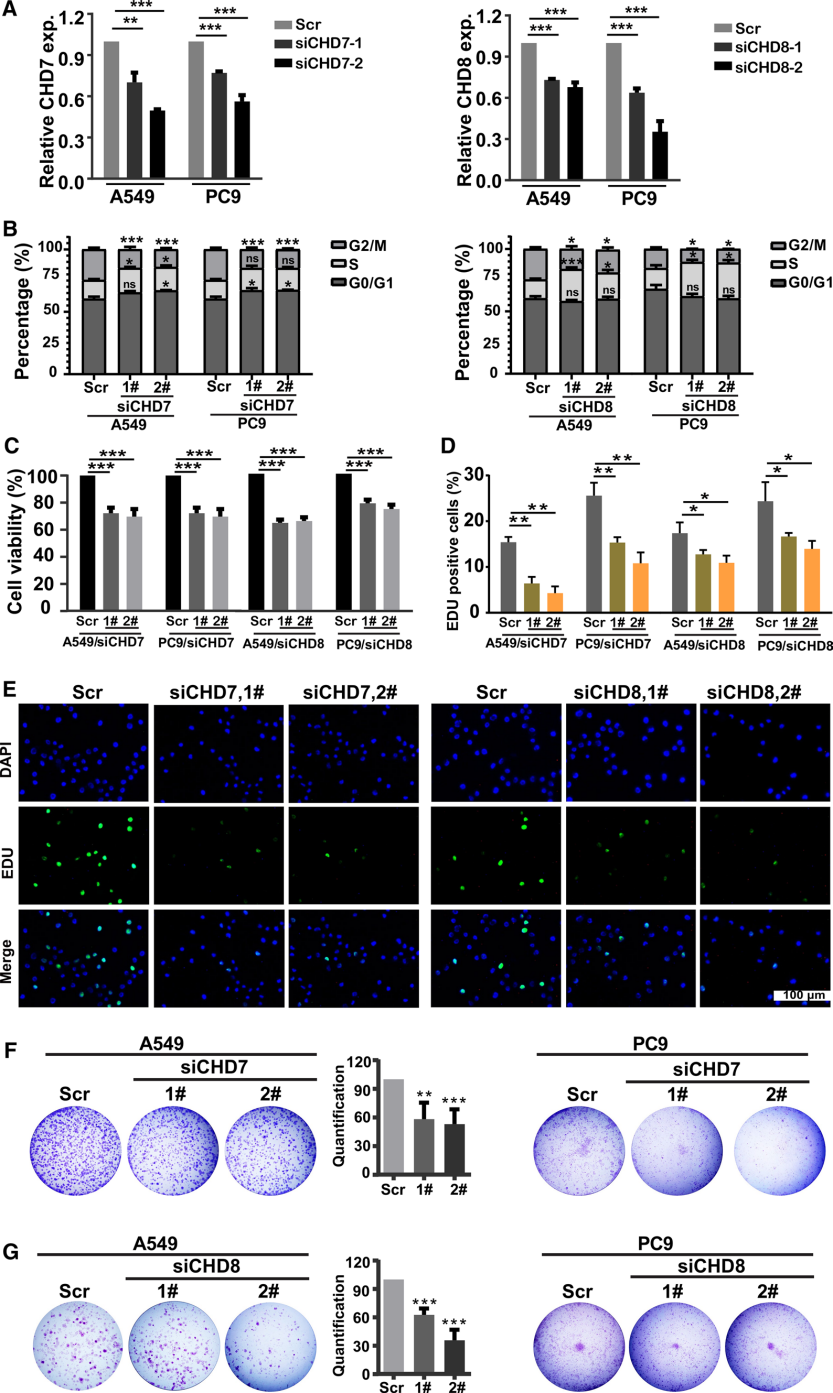
The effects of CHD7/8 knockdown on cell viability and cell cycle progression in lung adenocarcinoma. **A** RT–qPCR analysis showing efficient silencing of CHD7 (left panel) and CHD8 (right panel). **B** The effect of CHD7/8 depletion in A549 and PC9 cells on cell cycle progression. **C** The effect of CHD7/8 depletion in A549 and PC9 cells on cell viability. Cell viability was determined using the Cell Titer-Glo luminescent cell viability assay. **D** Quantification of EDU positive cells from EDU staining in A549 and PC9 cells with control, CHD7 or CHD8 knockdown.. E Representative images of EDU staining in PC9 cells with control, CHD7 or CHD8 knockdown. Scale bar, 100 μm.**F-G** Clonogenic assay of A549 and PC9 cells after knockdown of CHD7 (F) and CHD8 (G). Percent surviving fraction was calculated relative to nontreated cells. *p < 0.05, **p < 0.01, ***p < 0.001 (Student’s t test)

Because CHD7 and CHD8 were discovered as crucial genes among the CHD family members and were associated with the cell cycle and DNA repair in LUAD, we speculated that CHD7 and CHD8 play indispensable roles in cell cycle progression and the DNA damage response. To test this hypothesis, the expression of CHD7 and CHD8 was knocked down using siRNAs in A549 and PC9 cells (Fig. 6A). CellTiter-Glo luminescent assays revealed that cell viability was significantly inhibited upon knockdown of CHD7 and CHD8 (Fig. 6C). In addition, EDU staining assay suggested that CHD7 and CHD8 silencing could inhibit cell proliferation in both A549 and PC9 cells (Fig. 6D, 6E). Moreover, clonogenic assays showed that silencing of CHD7 and CHD8 significantly inhibited cell growth (Fig. 6F, 6G). First, we assessed the prognostic effects of CHD7 and CHD8 in the GSE3213 and MICHIGAN-LC databases, respectively, and the results further indicated that high expression of CHD7 (HR = 1.57, p < 0.05) or CHD8 (HR = 1.55, p < 0.05) may lead to a worse prognosis in LUAD patients (Fig. S2A, S2B). We then determined the knockdown of CHD7 and CHD8 on cell cycle progression by flow cytometic analysis. As a matter of fact, CHD7 and CHD silencing induced downregulation of G2/M, and decreased expression of CHD8 triggered S arrest (Fig. 6B). Taken together, the above results indicate that CHD7 and CHD8 are involved in cell proliferation and cell cycle progression.

### CHD7/8 loss impairs the CHK1 DNA damage response

**Fig. 7:**
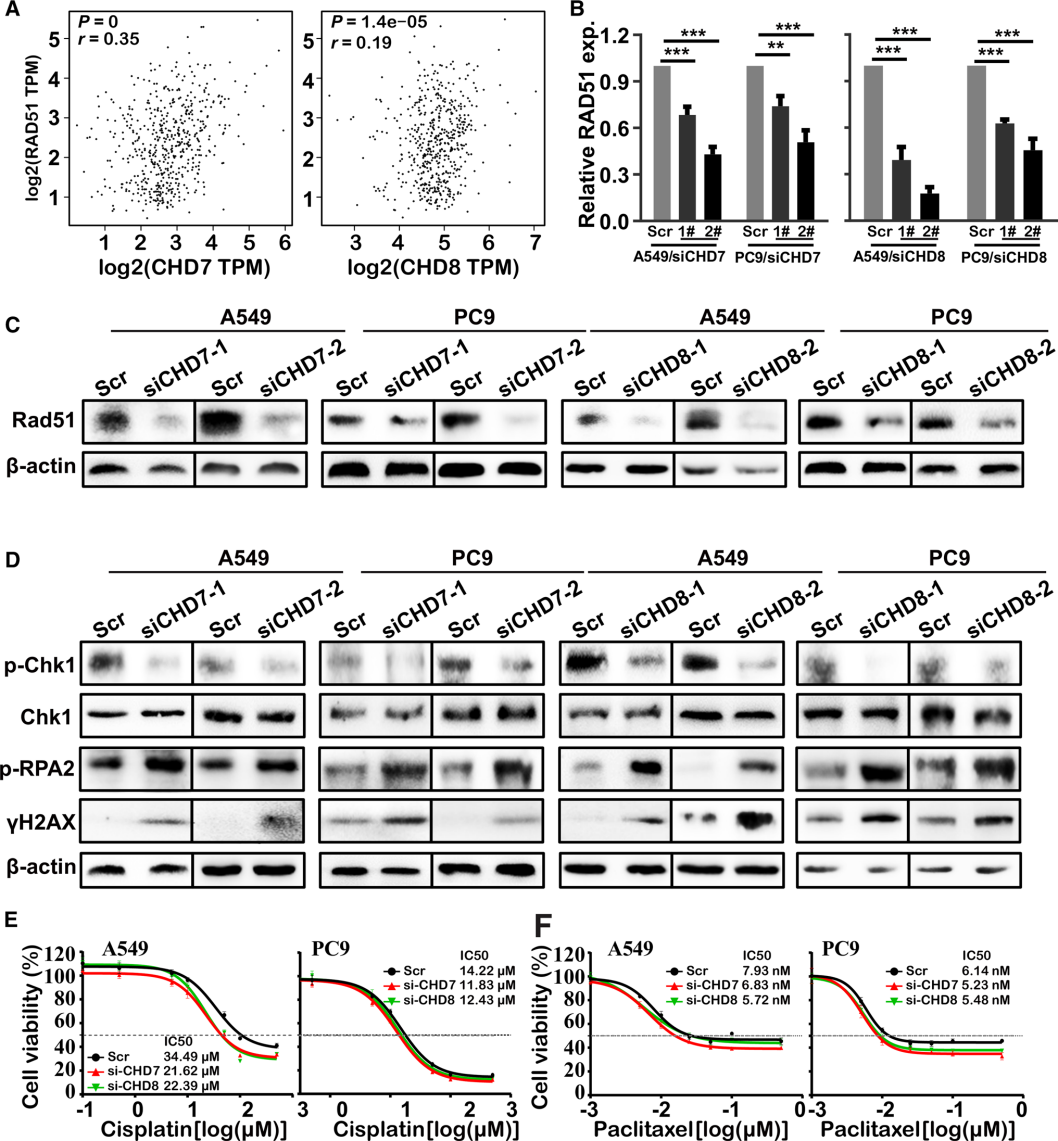
The effect of Chd7/8 knockdown on the DNA damage response in lung adenocarcinoma. A Pearson correlation analysis showing the correlation between CHD7/8 expression and RAD51 expression in LUAD; **B** RT–qPCR analysis of RAD51 upon CHD7 and CHD8 silencing; **C**, **D** Western blot analysis of Rad51 (C), γH2AX and p-chk1 (D) proteins upon depletion of CHD7 and CHD8; **E**, **F** Growth inhibition curves of cisplatin (**E**) and paclitaxel (**F**) in A549 and PC9 cells with CHD7 and CHD8 knockdown. The cell viability was assessed by the CellTiter-Glo assay. The IC50 values were determined from the sigmoidal dose–response curves using GraphPad PRISM6 software

We also assessed the effect of CHD7 and CHD8 knockdown on the DNA damage response. Western blot analysis of γH2AX, a classic DSB marker, demonstrated that γH2AX accumulated in CHD7 and CHD8 knockdown cells (Fig. 7D). We then aimed to explore the impact of the downregulation of CHD7 and CHD8 on DNA damage repair activity. The expression of RAD51, an essential recombinase in homologous recombination repair, was remarkably decreased at the mRNA and protein levels, when CHD7/8 was knocked down (Fig. 7B, C). Consistent with the western blot results, in the TCGA data analysis of LUAD, we found that the RNA expression of RAD51 was positively correlated with the RNA expression of CHD7/8 (Fig. 7A). Furthermore, p-CHK1 was significantly downregulated and p-RPA2 was upregulated after knocking down CHD7/8 (Fig. 7D). These observations suggest that CHD7/8 is involved in the regulation of DNA damage repair. To explore the chemosensitizing potential of targeting CHD7/8, we treated A549 and PC9 cells with a range of concentrations of cisplatin and paclitaxel to assess cell viability in the absence of CHD7/8. The results showed that either CHD7 or CHD8 silencing could enhance the cytotoxic activity of two commonly used chemotherapeutic drugs (cisplatin and paclitaxiel) (Fig. 7E).

## Discussion

High-throughput sequencing and biological experiments have established that CHD family members are associated with the occurrence and progression of a variety of cancer types. Although the functions of some members of CHDs have been discovered and demonstrated in lung cancer, systematic bioinformatics analysis of CHDs remains under investigation. The present study analyzed the expression, genetic alterations, prognostic values, and immune infiltrates of different CHD family members in non-small-cell lung cancer. Moreover, the predicted functions of CHD7 and CHD8 by KEGG were validated. Thus, our study provided the first systemic analysis of CHD family members in lung cancer as therapeutic targets for the diagnosis, prognosis, and treatment of non-small-cell lung cancer.

Our study showed that genetic alterations of CHDs are much less common in lung cancer than in gastric and colorectal cancers [12]. Among the nine CHD members, CHD7 is the most commonly mutated gene in lung adenocarcinoma and squamous cell carcinoma, with a mutation frequency of 6%. Additionally, CHD7 is the most commonly amplified in lung adenocarcinoma. In contrast, the expression of most CHD genes was frequently dysregulated. For example, in lung adenocarcinoma, CHD1/4/6/7/8 was significantly overexpressed, whereas CHD3 was downregulated. Compared with normal lung tissues, the expression of CHD4/6/7/8 in lung squamous cell carcinoma was higher, while the expression of CHD1/2, and to a less extent, was lower. Interestingly, we found that gene amplification and deletion might partially contribute to the dysregulation of those CHD genes. In addition, the results from our study demonstrated that the mRNA expression of CHD4/6/7/8 was remarkably linked with cancer stage in patients with lung adenocarcinoma, and CHD1/2/4/6/7/9 was associated with cancer stage in patients with lung squamous cell carcinoma. These different expression patterns and associations with tumor grades between adenocarcinoma and squamous cell carcinoma suggested that CHDs have a distinct effect on tumorigenesis in different tissues.

An accumulating body of literature indicates that the members of subclasses I and II function as tumor suppressors in a broad range of cancers [38–41]. In this study, the expression of CHD4 was higher in lung cancer than in normal tissues. However, low CHD4 expression was significantly correlated with poor OS and FP in all of the patients with lung adenocarcinoma, which seemed contradictory to the role of CHD4 as a tumor suppressor in other cancer types. Similar paradoxical phenomena also existed in lung squamous cell carcinoma, in which CHD1/2 was downregulated and its high expression was associated with poor OS and FP, and in lung adenocarcinoma, in which CHD3 was downregulated, its high expression was linked to poor OS and FP. More functional data are needed to further demonstrate and elucidate the roles of CHD4.

Unlike the subclasses I and II CHD proteins, subclass III CHDs have been poorly understood in lung cancer. An in-frame duplication of exons 3–7 of CHD7 and PVT1-CHD7 fusion has been identified in small cell lung cancer cells [42]. The results from our study showed that subclass III CHD genes appear to be strongly linked to both lung adenocarcinoma and squamous cell carcinoma. The expression of CHD7/8 in lung cancer tissues was higher than that in normal tissues. Furthermore, higher CHD7/8 expression was significantly correlated with poor OS and FP in all patients with lung adenocarcinoma and, to a lesser extent, lung squamous cell carcinoma. These observations indicate that CHD7/8 has an oncogenic function in lung cancer. These data highlight the critical roles of CH7/8 in lung cancer, especially in lung adenocarcinoma. Although CHD6 was overexpressed, its high expression level seems to be associated with a poor prognosis. The conflicting roles of CHD6 warrant further clarification.

Aberrant chromatin remodeling has also been found to be involved in the occurrence of cancer but also to impact the tumor immune environment. We first explored the correlations between CHD family genes and preimmune stages. The results indicated that the remaining genes, apart from CHD1 and CHD8, were strongly linked to preimmune stages in lung cancer. Next, we found that the mRNA expression levels of most CHD family genes were significantly positively correlated with the immune infiltrating levels of B cells, CD4 + T cells, CD8 + T cells, macrophages, neutrophils, and DCs. Therefore, among the 8 CHD members, CHD1 and CHD6 are likely to become targets of immunotherapy. This provides new ideas for subsequent research on lung cancer immunotherapy. Perturbation of CHD1/6 might remodel the tumor immune microenvironment and suggest that targeting CHD family members can be combined with immunotherapies in lung cancer in the future.

Furthermore, functional and pathway analysis predicted that CHD7/8 was mainly involved in the regulation of the cell cycle, DNA repair signaling, and other pathways. Subsequent in vitro experiments using PC9 and A549 cells confirmed that silencing CHD7/8 caused cell cycle arrest and attenuated DNA repair capacity based on flow cytometic analysis and evaluation of several proteins involved in DNA repair signaling.

There are some limitations to this study. First, our experimental data showed the functional connection between CHD7/8 and the DNA damage response pathway. However, the underlying mechanisms leading to the down-regulation of RAD51 and CHK1 phosphorylation remain to be determined. Second, functional enrichment analysis indicates that most CHD family genes, if not all, play essential roles during cell cycle progression and DNA repair. Nevertheless, the functional validation experiments were only performed for CHD7 and CHD8, but not for other CHD family genes in this study. It would be interesting to evaluate the effects of other CHD genes on these two cellular events in the future. Finally, the experimental procedures could be employed to validate the correlation between CHD genes and immune infiltrates and provide mechanistic insights into CHD-mediated immune infiltration.

In conclusion, we aimed to understand the clinical significance of the CHD family in lung cancer and the molecular mechanism based on big data. We systematically analysed the expression, genetic variations, and prognostic value of CHDs in lung cancer. Our study demonstrated that altered expression of some CHD members was significantly correlated with clinical cancer stages in lung cancer patients. Furthermore, our results indicated that high CHD6/7/8 expression could also serve as a promising prognostic indicator. CHD subclass III members play critical roles in the cell cycle DNA damage response and are potential epigenetic therapeutic targets.

## Supplementary Information

Additional file1 (PDF 1558 KB)

Additional file2 (XLSX 13 KB)

## Data Availability

The datasets generated during and/or analysed during the current study are available in the TCGA repository, https://tcga-data.nci.nih.gov/tcga; MEXPRESS repository, https://mexpress.be; Oncomine repository, https://www.oncomine.org; UALCAN repository, http://ualcan.path.uab.edu; Human Protein Atlas repository, https://www.proteinatlas.org; cBioPortal repository, https://www.cbioportal.org; CR2Cancer repository, http://cis.hku.hk/CR2Cancer; KMplot repository, https://.kmplot.com; Timer repository, https://cistrome.shinyapps.io/timer; TISIDB repository, http://cis.hku.hk/TISIDB; Metascape repository, http://gepia.cancer-pku.cnhttps://metascape.org

## Abbreviations

CHDs: Chromodomain helicase DNA-binding family
LUAD: Lung adenocarcinoma
LUSC: Lung squamous cell carcinoma
CNV: DNA copy number variation
ROC: Receiver operating characteristic
AUC: Area under the curve
HR: Hazard ratio
OS: Overall survival
FP: Progression survival
CBXs: Chromobox family
