# Supplementary material for: Comprehensive analysis of the expression, prognosis, and immune infiltrates for CHDs in human lung cancer
Source: Discov Oncol. 2022 Apr 25;13:29. doi: 10.1007/s12672-022-00489-y (PMC9038980; doi:10.1007/s12672-022-00489-y)
Supplement: Supplementary file 1 — Additional file1 (PDF 1558 KB) [file 12672_2022_489_MOESM1_ESM.pdf]

## Supplementary Files

# Comprehensive analysis of the expression, prognosis, and immune infiltrates for CHDs in human lung cancer

Yang Lv[1,2,3,], Wenchu Lin[1,3]*

[1]High Magnetic Field Laboratory, Hefei Institutes of Physical Science, Chinese Academy of Sciences, Hefei 230031, Anhui, P. R. China

[2]University of Science and Technology of China, Hefei 230026, Anhui, P. R. China

[3]Key Laboratory of High Magnetic Field and Ion Beam Physical Biology, Hefei Institutes of Physical Science, Chinese Academy of Sciences, Hefei 230031, Anhui, P. R. China

***Corresponding Author:**

Wenchu Lin, Ph.D

High Magnetic Feld Laboratory, Hefei Institutes of Physical Science, Chinese Academy of Sciences

Tel: 86-551-65593499, E-mail: wenchu@hmfl.ac.cn.

**Running title**: The roles of CHDs in lung cancer

**Competing interests**

The authors declare no competing interests

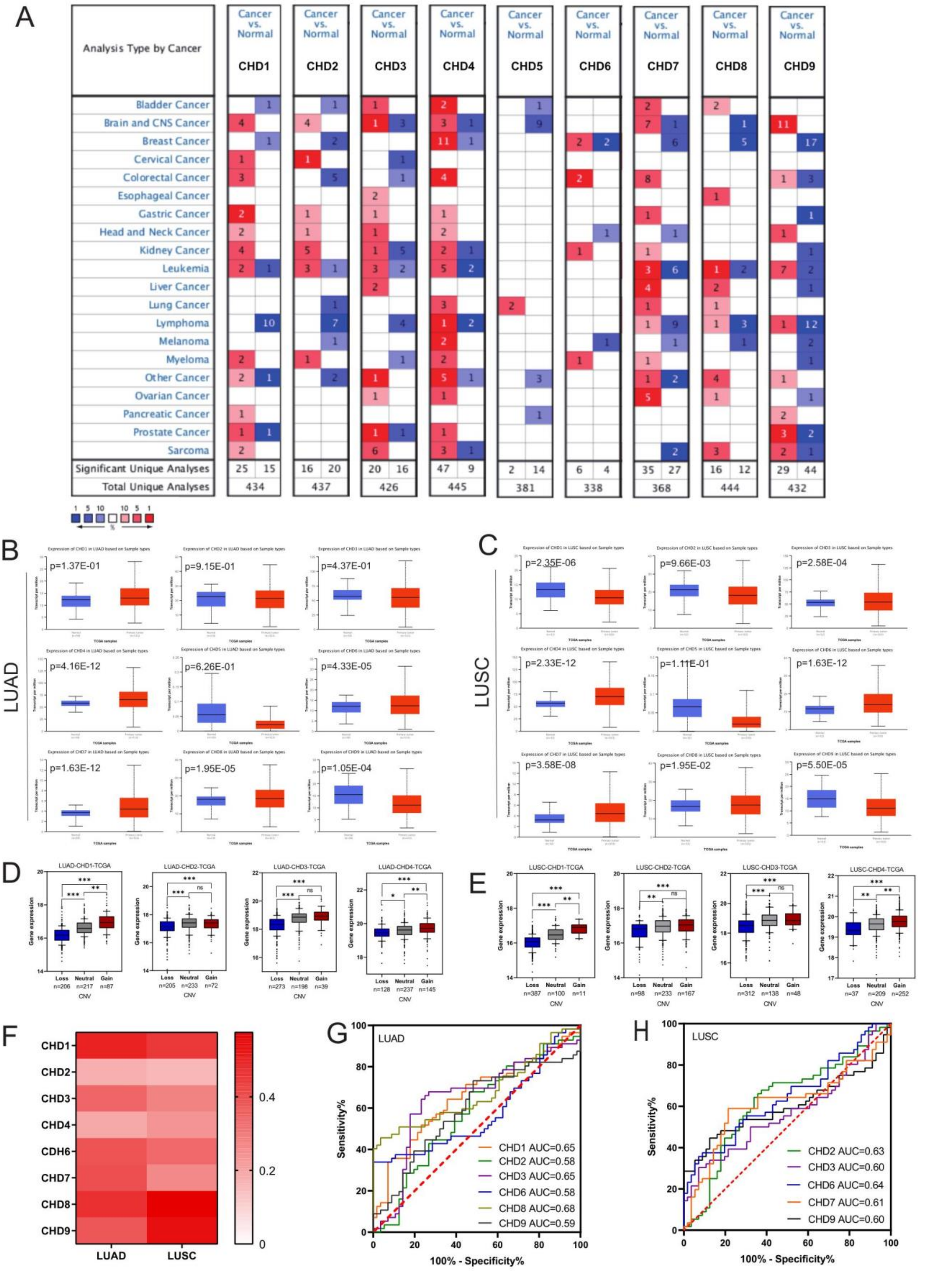


**SF.1 The transcription levels of CHDs factors in different types of cancers (Oncomine). A)** The panel showing the

numbers of datasets with statistically significant mRNA up-expression (red) or downregulated expression (blue) of CHDs in different types of cancer versus normal tissues. The threshold was designed with the following parameters: fold change of 1.5 and P-value of 0.01; **B-C)** Box plot showing the distribution of the mRNA expression of CHDs across LUAD (B) or LUSC (C) tissues and adjacent normal tissues (UALCAN); **D-E)** box plot showing the correlation of copy number variation and CHD expression in LUAD (D) and LUSC (E); **F)** In heatmap, shades of red or white represent the correlation values between copy number variation and CHD expression in lung cancer; **G-H)** ROC analysis of CHD expression for distinguishing lung cancer from normal lung tissues. *$p<0.05$, **$p<0.01$, ***$p<0.001$ (Student's t test).

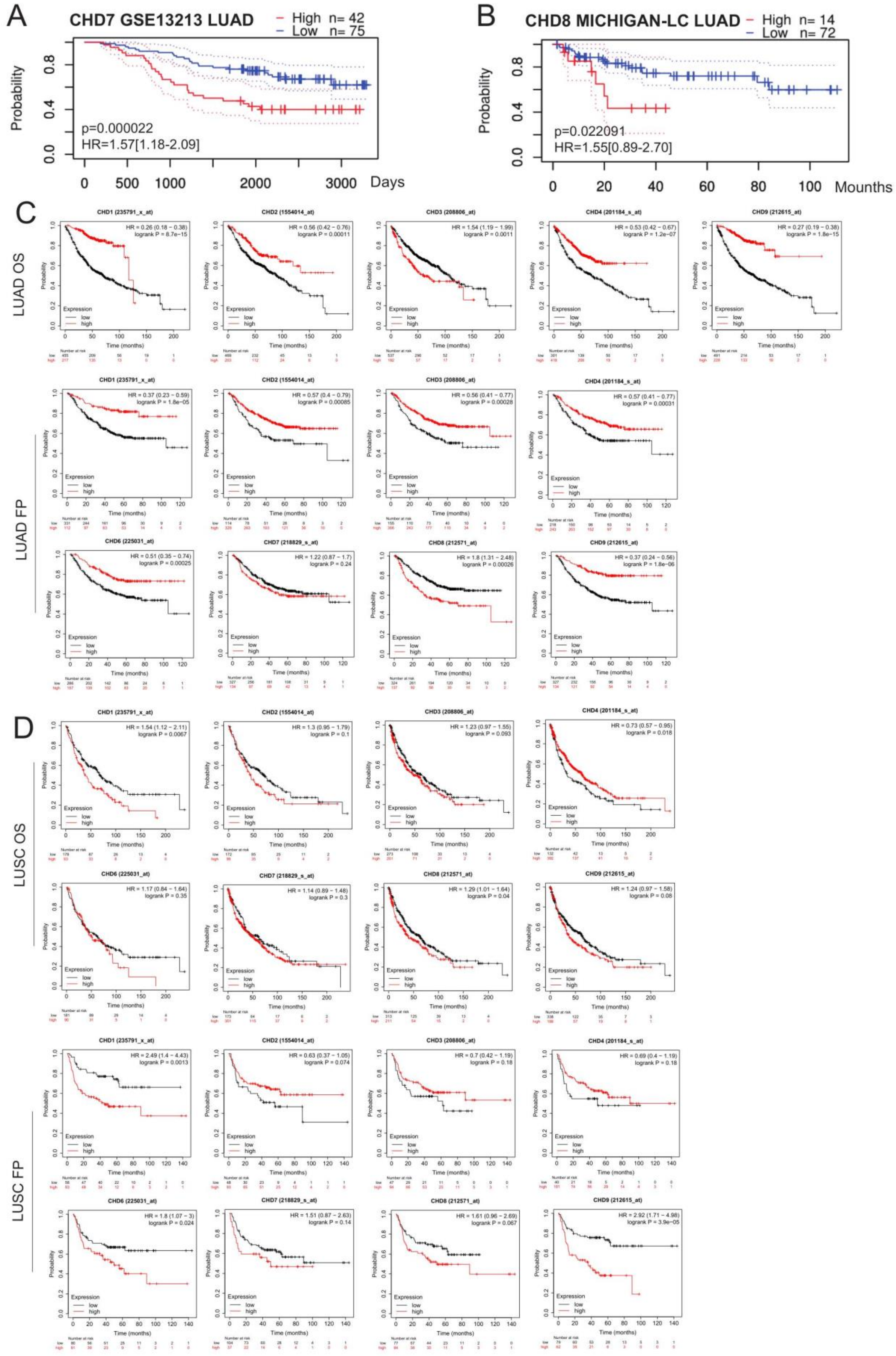
A
CHD7 GSE13213 LUAD
High n= 42
Low n= 75
Probability
p=0.000022
HR=1.57[1.18-2.09]
Days
B
CHD8 MICHIGAN-LC LUAD
High n= 14
Low n= 72
Probability
p=0.022091
HR=1.55[0.89-2.70]
Mounths
C
LUAD OS
LUAD FP
D
LUSC OS
LUSC FP

**SF.2 The prognostic value of CHDs in LUAD and LUSC patients. A-B)** Kaplan–Meier analysis showing that expression of CHD7/8 was linked to overall survival in LUAD based on the two cohorts (GSE13213 and MICHIGAN-LC); **C-D)** Kaplan–Meier analysis showing that expression of CHDs was associated with overall survival and first progression survival in LUAD and LUSC.

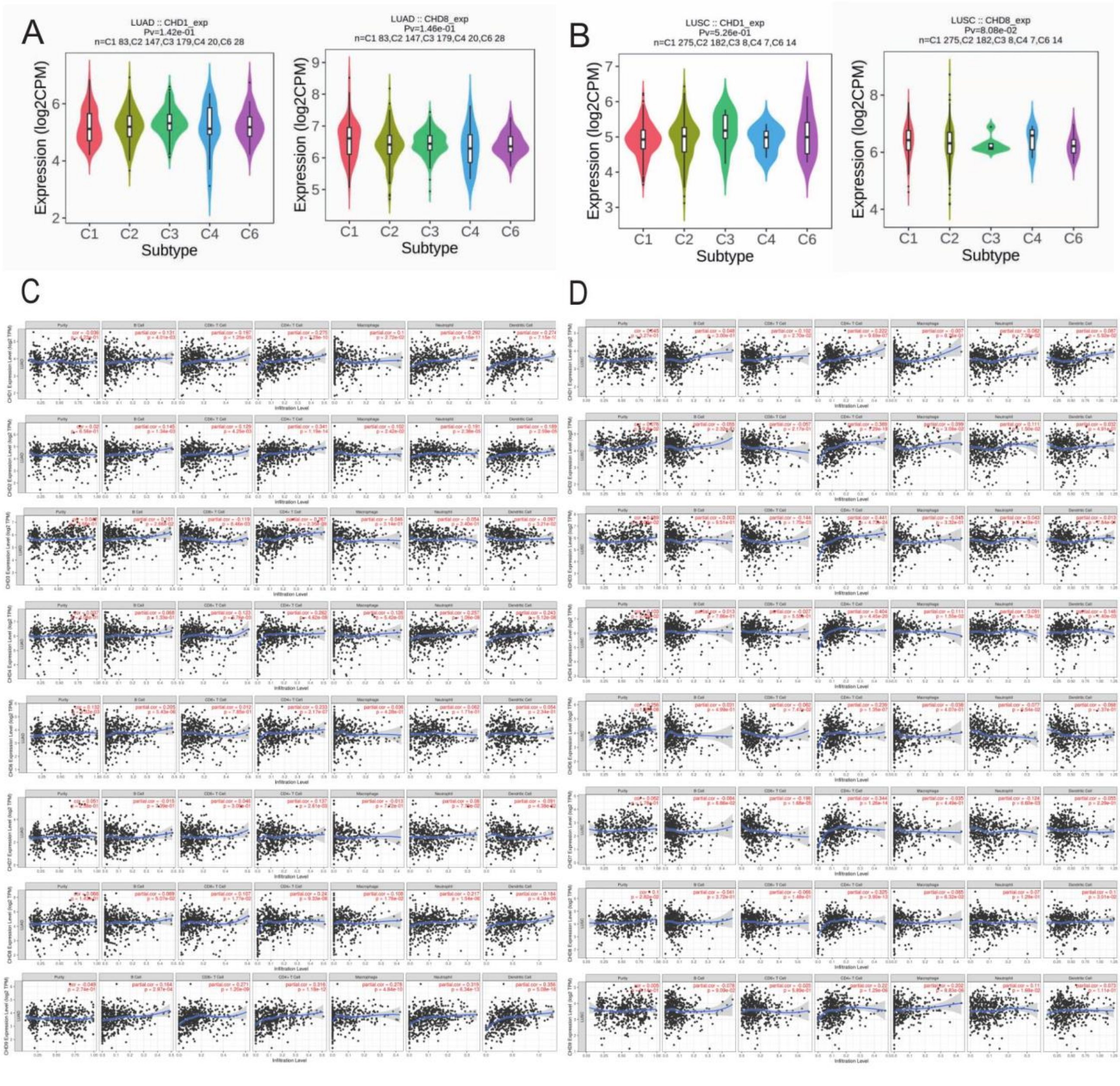


**SF.3 Immune features of CHDs had no significance. A-B)** The relationship between CHD expression and immune subtypes in LUAD (A) and LUSC (B); **C-D)** CHD expression had significant positive correlations with infiltrating levels of B cells, CD8+ T cells, CD4+ T cells, macrophages, neutrophils, and dendritic cells in LUAD and LUSC.

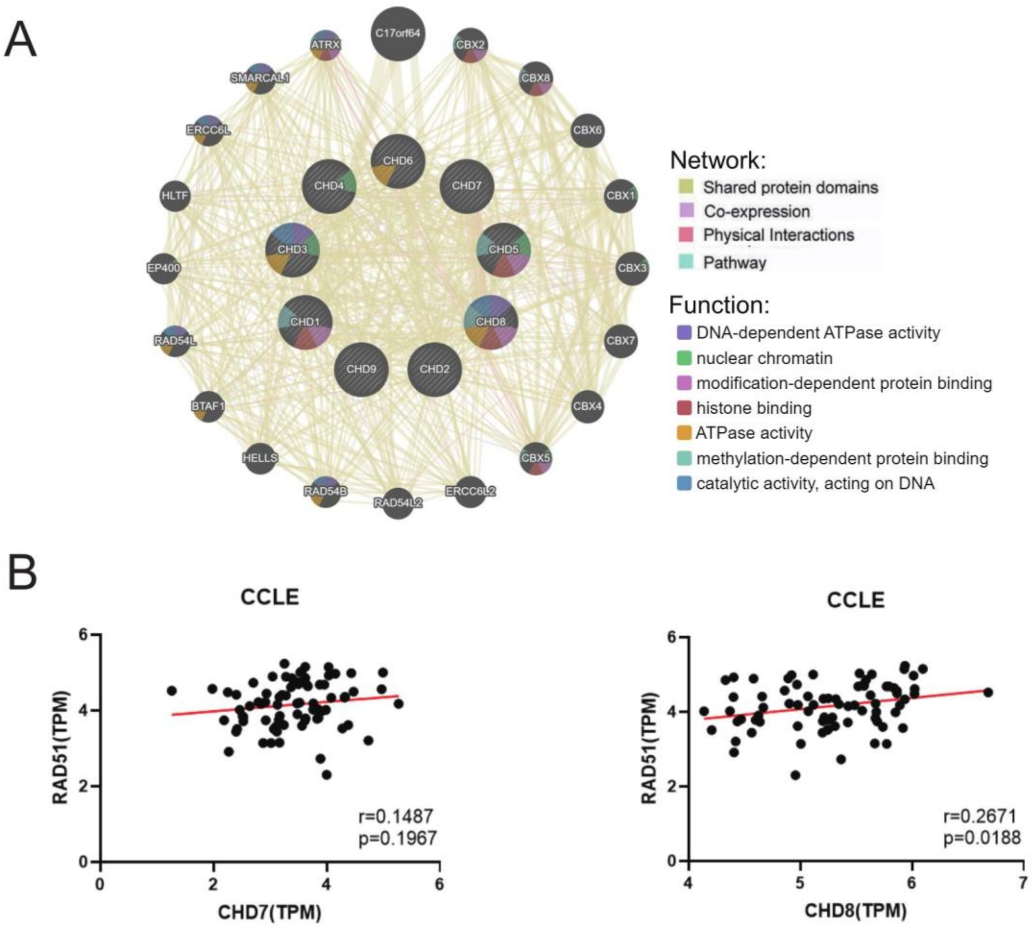


**SF.4 Functional enrichment analysis of CHDs in patients with lung cancer. A)** Gene–gene interaction network of different expressed CHDs. Each node represents a gene, and the size of the node represents the strength of the interaction. The color of the connection lines between nodes represents the type of gene–gene interaction. Node colors indicate the possible functions of the respective genes; **B)** Pearson correlation analysis showing the correlation between CHD7/8 expression and RAD51 expression in LUAD cell line and LUSC cell lines.

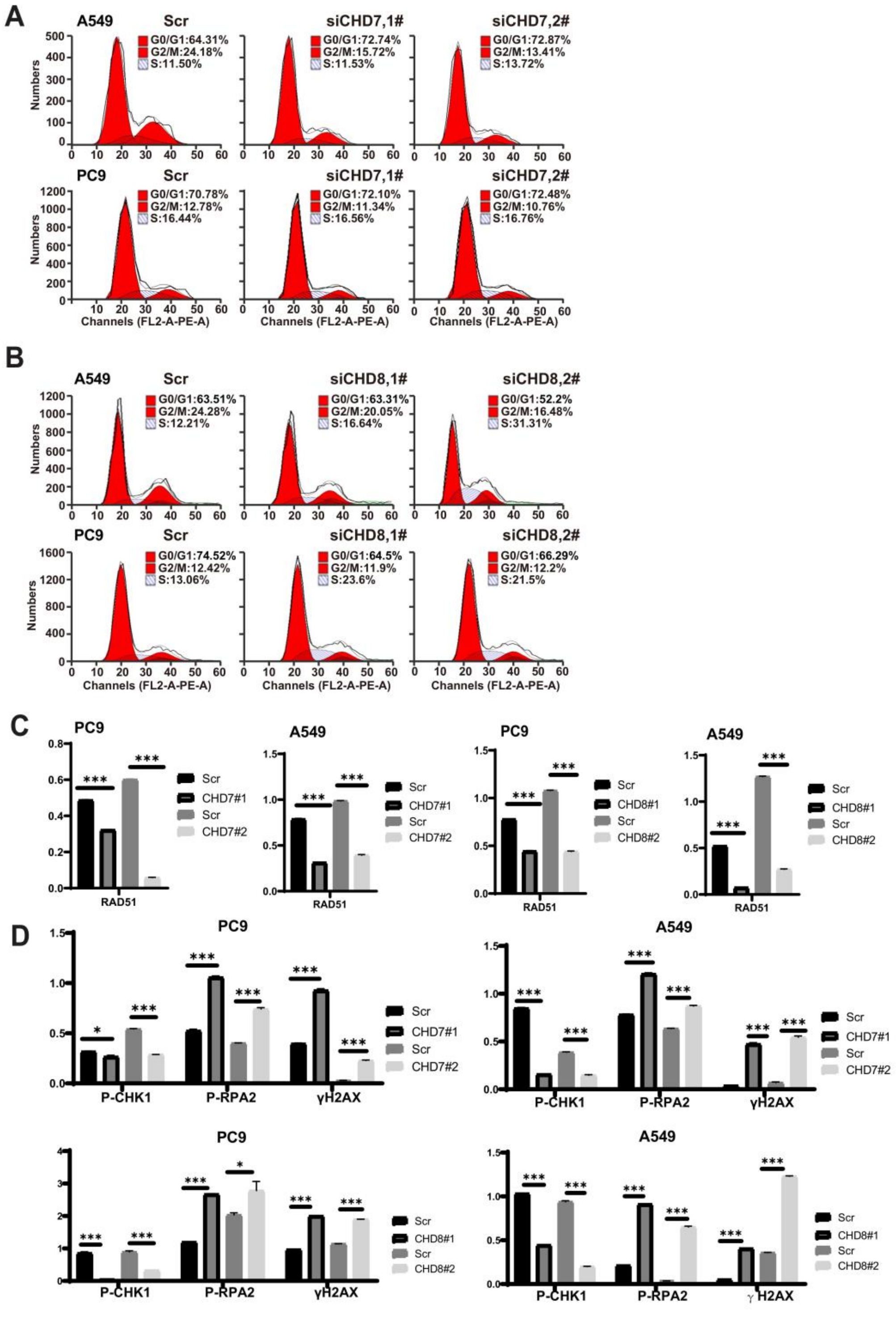


**SF.5 The flow cytometry graph and the quantification data. A-B)** flow cytometry graph of figure 6B; **C)** Densitometry analyses for RAD51 in figure 7C; **D)** Densitometry analyses for p-CHK1, p-RPA2 and γH2AX in figure 7D. $*p<0.05$, $**p<0.01$, $***p<0.001$ (Student's t test).
